# Nanoemulsions of sulfonamide carbonic anhydrase inhibitors strongly inhibit the growth of *Trypanosoma cruzi*

**DOI:** 10.1080/14756366.2017.1405264

**Published:** 2017-12-01

**Authors:** Alane Beatriz Vermelho, Verônica da Silva Cardoso, Eduardo Ricci Junior, Elisabete Pereira dos Santos, Claudiu T. Supuran

**Affiliations:** a Bioinovar-Biotecnologia: Unidade de Biocatálise, Bioprodutos e Bioenergia (BIOINOVAR), Instituto de Microbiologia Paulo de Góes, Universidade Federal do Rio de Janeiro, Rio de Janeiro, RJ, Brazil;; b Laboratório de Desenvolvimento Galênico (LADEG), Departamento de Medicamentos, Universidade Federal do Rio de Janeiro, Rio de Janeiro, RJ, Brazil;; c NEUROFARBA Dept., Sezione di Scienze Farmaceutiche, Università degli Studi di Firenze, Florence, Italy

**Keywords:** Carbonic anhydrase, *Trypanosoma cruzi*, nanoemulsion, sulfonamide, Chagas disease

## Abstract

Sulfonamide carbonic anhydrase (CA, EC 4.2.1.1) inhibitors targeting the α-class enzyme from the protozoan pathogen *Trypanosoma cruzi,* responsible of Chagas disease, were recently reported. Although many such derivatives showed low nanomolar activity *in vitro*, they were inefficient anti-*T. cruzi* agents *in vivo*. Here, we show that by formulating such sulfonamides as nanoemulsions in clove (*Eugenia caryophyllus*) oil, highly efficient anti-protozoan effects are observed against two different strains of *T. cruzi*. These effects are probably due to an enhanced permeation of the enzyme inhibitor through the nanoemulsion formulation, interfering in this way with the life cycle of the pathogen either by inhibiting pH regulation or carboxylating reactions in which bicarbonate/CO_2_ are involved. This type of formulation of sulfonamides with *T. cruzi* CA inhibitory effects may lead to novel therapeutic approaches against this orphan disease.

## 1. Introduction

Chagas disease, caused by the protozoan *Trypanosoma cruzi*, is endemic in regions of Central and South America. In Latin America, five to eight million people are infected with this protozoan^1^. Infection starts by blood-sucking triatomine bugs, but others transmissions routes are possible, such as organ transplantation, congenital contamination, blood transfusion as well as contaminated foods and drinks^2^^,^^3^. Due to these modes of transmission the disease is spreading to nonendemic countries including Australia, Canada, Japan, Spain, and the USA^2^^,^^3^. The drugs used for the treatment of the disease are the nitroheterocyclic compounds benznidazole and nifurtimox but both of them induce severe side effects and cross-resistance^4^. New therapeutic approaches and new drugs are investigated constantly, and in the present work, nanoemulsions (NEs) of sulfonamides derivatives with inhibitory effects against the carbonic anhydrase (CA, EC 4.2.1.1) from *Trypanosoma cruzi* were developed.

NEs have been widely used in the pharmaceutical area as drug carriers. Due to the existence of polar and apolar phases at the interfacial domain, NEs are versatile release systems able to encapsulate drugs with variable solubility^5^. The majority of NEs are dispersions of oil droplets in water with diameter between 20 and 200 nm. NEs present small droplet size that allows the Brownian motion of the drops retarding their sedimentation or coalescence. Thus, NEs present kinetic stability^6^, promoting tissue permeation and penetration of drugs. Their nanometric droplets have large relative surface area, facilitating the contact of the nanocarrier with the biological membrane or tissue, and consequently favouring drug permeation and retention. The surfactants included in the NEs can promote reduction of the surface tension between the droplets and biological tissue or membrane, improving the drug’s spreadability and bioadhesion. These droplets can also act as a reservoir system for sustained drug release. Moreover, the main advantages of nanocarriers are the ease of preparation, possibility of industrial-scale production, and high thermodynamic stability^7^.

NE preparations have been used to improve drug activity. 2-(Butylamino)-1-phenyl-1-ethanethiosulfuric acid (BphEA) is a promising schistosomicidal drug; however, it presents low solubility in water and low effectiveness against the parasite. The NEs containing BphEA were produced utilizing ultrasound, oil phase with medium-chain triglycerides (coconut oil) and stearylamine, and mixtures of nonionic surfactants (Span 80 and Tween 80). The drug in NE form presented more schistosomicidal activity than the solution. The NE interacted with the surface membrane of the parasite promoting the permeation of the drug and schistosomicidal activity^8^. Zinc phthalocyanine (ZnPc) and chloroaluminum phthalocyanines (ClAlPc) are photosensitizers used in photodynamic therapy of cancer. However, these photosensitizers present water solubility problems. NEs were used to solve the solubility problems leading to enhanced effectiveness of these photosensitizers. The NEs containing ZnPc or ClAlPc were produced utilizing ultrasonic processor, oil clove, and nonionic surfactants (Lutrol^®^ F-68). The results showed that the photosensitizers in the NE form were more affective in the elimination of tumour cells (cells A549, human lung carcinoma cells) than the photosensitizer solution alone^9^.

Carbonic anhydrase (CA, EC 4.2.1.1) inhibition has pharmacologic applications in various fields, with antiglaucoma^10^, diuretics^11^, antiepileptics^12^, antiobesity^13^, and antitumour agents^14^ belonging to various classes of such pharmacological agents (sulfonamides, coumarins, dithiocarbamates, etc.)^10–14^. Recently, the potential use of CA inhibitors (CAIs) as anti-infectives also started to be considered, with antibacterials^15–17^, antifungals^18^^,^^19^, and antiprotozoan agents^20–22^, being investigated in the search for agents devoid of the resistance problems common to most classes of clinically used such agents^23^. We have, for example, reported that *T. cruzi*, the etiological agent of Chagas diseases^20^ encodes for an α-CA, called TcCA^20^. This enzyme was inhibited *in vitro* by many sulfonamides in the low nanomolar or subnanomolar range^24^^,^^25^. However, *in vivo*, the growth of the parasite was not inhibited by such sulfonamides^24^^,^^25^. Only some heterocyclic thiols^20^ and hydroxamates^26^^,^^27^ did show *in vivo* efficacy as anti-*T. cruzi* agents (and they also acted as efficient *in vitro* TcCA inhibitors)^24–28^, and we considered that this might be due to the lack of permeability of the sulfonamides through the biological membranes of the protozoan. This is the reason why we decided to investigate the formulation of such sulfonamides, highly effective as TcCA inhibitors in NEs, in order to enhance their bioavailability and penetrability through membranes. Here, we report that sulfonamide TcCA inhibitors formulated as NEs in clove oil, potently inhibit the growth of *T. cruzi ex vivo*, showing thus a potential as a novel class of antitrypanosomal drugs.

## 2. Materials and methods

### 2.1 Chemistry

Sulfonamides **3F**, **3G**, **3W**, **5B**, **5C**, and **5D** used in the experiments were reported in an earlier work from our groups^21^.

### 2.2 Materials

Clove oil (*Eugenia caryophyllus*) was purchased from Ferquima Ltd. (Brazil). Pluronic F-127, a nonionic block-copolymer surfactant of (poly(ethylene oxide)-block-poly(propylene oxide)-block-poly(ethylene oxide)) (EO_100_PO_66_EO_100_), with MW 12,600, and HLB 22, was purchased from Sigma Aldrich (Milan, Italy). Dulbecco’s modified Eagle’s medium (DMEM), resazurin, Benznidazol (Bz): 2-nitro-imidazole-(N-benzil-2-nitro-1imidazoleacetamide), and thiazolyl blue tetrazolium bromide (MTT) were purchased from Sigma-Aldrich. Fetal bovine serum (FBS) was purchased from LGC Biotecnologia (São José, Cotia, Brazil).

### 2.3 NE preparation

**Figure 1.**
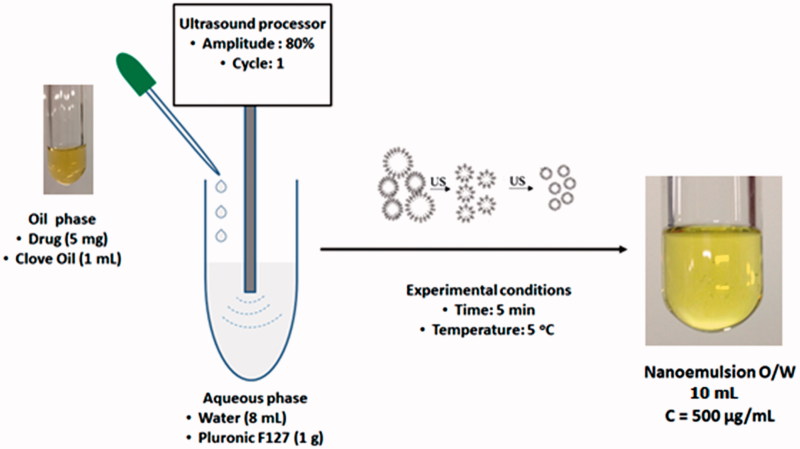
Preparation of nanoemulsion by high-energy method.

The oil-in-water (O/W) NEs were prepared by high-energy method (Figure 1) using an ultrasound processor (Hielscher model UP100H), according to a method adapted from literature^29^. Oil phase was prepared by sulfonamides dissolution in the clove oil. A 5 mg of drug was weighted in an eppendorf and 1 ml of clove oil was added. The tube was agitated for 1 min for obtaining of the drug solution (5 mg/ml). Aqueous phase were prepared by adding 1 g of Pluronic F127^®^ in 8 mg of water. Then 1 ml of oil phase (drug dissolved in clove oil) was added to 9 ml of aqueous phase (Pluronic F127 in water) under constant ultrasound homogenization (amplitude 80%, continuous cycle n. 1) during 5 min in an ice bath at 5 °C to prevent heating of the dispersion. A transparent NE was obtained at a concentration of 500 µg/ml (Figure 1).

### 2.4 Determination of droplet size

Determination of droplet size and polydispersity index (PDI) were measured using the dynamic light scattering (DLS) method with a Malvern model 90S NanoSizer^®^ (UK). NEs were diluted in distilled water at 1:10 and analyzed in a cell with 1 cm optical path at room temperature (25 °C). These analyses were conducted in three runs with fifteen readings. The values shown are the mean ± standard deviation of three measurements for each formulation. The PDI reflects the sample quality in the parameter homogeneity of the droplet diameter. PDI results lower than 0.3 were considered satisfactory^30^.

### 2.5 T. cruzi parasites

Epimastigote forms of the *T. cruzi* clone Dm28c (lineage TCII)^31^ and Y(lineage TCI)^32^^,^^33^ strains obtained from the Laboratory of Cellular Ultrastructure (both laboratories of the Oswaldo Cruz Foundation, Rio de Janeiro, Brazil) were used. The parasites were maintained in PBHIL medium supplemented with 10% bovine serum (FBS) at 28 °C^34^.

### 2.6 RAW 264.7 macrophage cell line culture

RAW 264.7 macrophages were obtained from the National Institute of Metrology, Quality and Technology (Instituto Nacional de Metrologia, Qualidade e Tecnologia, INMETRO) and maintained in DMEM medium supplemented with 10% FBS at 37 °C in a 5% controlled CO_2_ atmosphere. Cell maintenance was performed every 48–72 h, time necessary for cells to achieve confluent monolayers.

### 2.7 Evaluation of NEs activity on T. cruzi epimastigotes

The evaluation of NEs antiprotozoal activity was performed by successive microdilutions in 96 well plates (1.8 × 10^6^ parasites/well) NEs in the PHBIL medium supplemented with 10% FBS in the following concentrations: 128, 64, 32, 16, 8, 4, 2, and 1 μM. The experiment controls were: negative control (culture medium with parasite) and positive culture (culture medium with parasite), and Benznidazole (as reference drug) was also progressively diluted with the parasite. The Minimum inhibitory concentration (MIC) for epimastigotes was performed with resazurin as an indicator of cellular metabolic function and it was determined as the lowest concentration capable of inhibiting *in vitro* growth of the parasites^35^. The determination of IC_50_ and IC_90_ (concentration of drug that reduces epimastigotes proliferation by 50%) was obtained by distance from the line from the inhibition values (%).

### 2.8 Cytotoxicity essay in macrophages

Sulfonamide NEs cytotoxicity was performed using tetrazolium dye (MTT) colorimetric assay. RAW 264.7 macrophages were harvest after confluent monolayer achievement. The cells were washed twice with PBS and a cellular suspension of 10^6^ cells/ml was prepared in fresh DMEM culture medium. Aliquots of 100 µl of the cellular suspension were placed into polystyrene 96-well plates, and then incubated at 37 °C in a 5% CO_2_ atmosphere for 6 h to allow for adherence of macrophages. After this period, the adherent cells were subjected to treatment with several concentrations of the sulfonamide NEs (1–128 µM), and then incubated for additional 48 h. Finally, 20 µl of a MTT solution (5 mg/ml) were added to each well and the plates incubated for 4 h^36^. Macrophage viability was determined after formazan crystals solubilization with DMSO followed by the absorbance measurement at 570 nm using a SpectraMax M5 spectrophotometer (Molecular Devices, Sunnyvale, CA).

### 2.9 Determination of selectivity index

A selectivity index (SI) was calculated and is defined as the RAW IC_50_ value divided by the *T. cruzi* IC_50_ value (IS = CC_50_/IC_50_), which expresses the safety index of the tested substance^37^. Benznidazole (Sigma-Aldrich, Milan, Italy) was kept as a positive control drug for the cytotoxicity assay on RAW 264.7.

### 2.10 Flow cytometry

The analysis of the externalization of phosphatidylserine was performed using annexin V fluoresceine isothiocyanate (AV FITC) and propidium iodide, PI (Santa Cruz Biotechonology, Santa Cruz, CA). The cells were analysed by flow cytometry (FACS Calibur-Beckton-Dickinson), 50,000 events were analysed by the Paint-a-gate program and values were expressed as percentage of cells positive for a given marker relative to the total number of cells: FITC-labelled cells (viable cells), PI-labelled cells (apoptotic cells), and cells with ruptured cell membrane (necrotic cells will be double-labelled).

### 2.11 Statistical analysis

The data of the experiments are being carried out through the program Prism 5.01 GraphPad (GraphPad Software, Los Angeles, CA), being considered values statistically significant those with values *p* < 0.05. The values were expressed as the mean ± standard deviation (SD). The analysis of variance was done by the Student–Newman–Keuls test for comparison between means.

## 3. Results and discussion

### 3.1 Preparation of sulfonamide NEs

**Figure 2.**
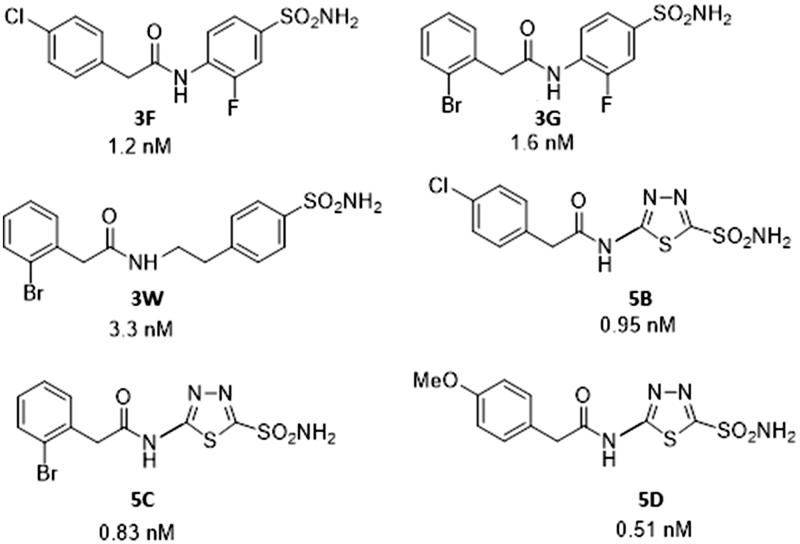
Sulfonamides **3F**, **3G**, **3W**, **5B**, **5C**, and **5D** used in the study and their TcCA inhibitory action.

Drugs **3F**, **3G**, **3W**, **5B**, **5C**, and **5D** act as highly efficient TcCA inhibitors *in vitro*^25^, with inhibition constants ranging between 0.51 and 3.3 nM (Figure 2). However, as mentioned above, they did not show *in vivo* anti-trypanosomal effects^25^. This is the reason why they were formulated as NEs in clove oil.

Sulfonamides **3F**, **3G**, **3W**, **5B**, **5C**, and **5D** were dissolved efficiently in clove oil in the concentration de 5 mg/ml. The NEs were produced with 10% of oil phase. NEs were prepared also without the drug, in order to evaluate the stability, droplet size, and PDI. The NEs obtained were yellow and transparent suggesting that the system was homogeneous with small droplet size (Table 1). As phase separation and precipitation of the drug were not observed, the NEs were considered stable in the concentration of 500 µg/ml.

**Table 1.** NEs size and polydispersity index

Formulation/drug	Drug (mg)	Oil Clove (ml)	AP (ml)	Size (nm)	PDI	Stability
NE	–	1	9	31.54 ± 0.413	0.105 ± 0.012	Stable
NE-3F	5	1	9	60.12 ± 2.36	0.274 ± 0.033	Stable
NE-3G	5	1	9	100.63 ± 2.05	0.262 ± 0.008	Stable
NE-3W	5	1	9	97.34 ± 2.82	0.264 ± 0.15	Stable
NE-5B	5	1	9	44.83 ± 0.753	0.123 ± 0.078	Stable
NE-5C	5	1	9	53.99 ± 1.12	0.233 ± 0.003	Stable
NE-5D	5	1	9	35.09 ± 0.575	0.165 ± 0.019	Stable

AP: aqueous phase containing tensioactive (Pluronic F127) and water, drug concentration 500 µg/mL, mean ± SD (from three different determinations).

NEs without the drug presented an average size of 31.54 nm. The NEs containing drug presented average sizes between 35 and 100 nm, depending on the drug. The lowest average size was exhibited by NE **5D** with 35.09 nm. NE-**3G** and NE-**3W** exhibited the larger average size values with 100.63 and 97.34 nm, respectively. The NEs presented PDI below 0.3, indicating that the size distribution is homogeneous and monomodal. Thus, we conclude that the inclusion method of the drugs in NEs was adequate producing nanostructured samples with drops below 100 nm and size distribution homogeneous and monomodal.

### 3.2 Anti-T. cruzi activity *in vivo*

**Figure 3.**
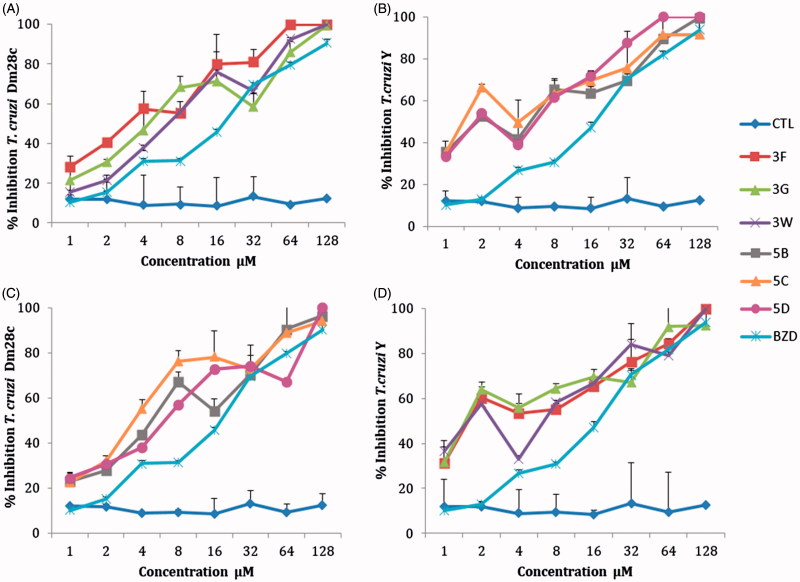
Inhibition effects of different concentrations of nanoemulsions with sulfonamide derivatives (1–128 μM) **3F**, **3G**, **3W**, **5B**, **5C**, **5D**. (A and C) Epimastigotes *T. cruzi* Dm28c strain; (B and D) epimastigotes *T. cruzi* Y strain, after 5 days of incubation. CTL control nanoemulsions without sulfonamide derivatives, BZD: benznidazole reference drug (1–128 μM).

The percentage inhibition of epimastigote forms at different concentrations of NEs are shown in Figure 3. The sulfonamide **3F** in a concentration of 4 µM inhibited 57% and 43.51% the epimastigotes forms of DM28c and Y strain of *T. cruzi,* respectively. The **5C** derivative showed a significant inhibition of 55.2% (for DM28c strain) and of 49.4% (Y strain) at 4 µM concentration of drug.

The half maximal inhibitory concentration (IC_50_) values of the sulfonamides NEs were lower than the benznidazol (20.63 µM) for the epimastigote forms of both strains of *T. cruzi*. (Y and DM28c). With the sulfonamides concentrations used, the MIC was >128 except for the sulfonamide **5D** for the *T. cruzi* Y strain (with a value of 64 µM). The sulfonamide **3F** showed the best activity with an IC_50_ of 3.54 µM. All derivatives showed cellular toxicity against macrophages cells RAW 267.4. A SI in the range of 1–3 was found for most of the sulfonamide inhibitors in NEs, when compared with the reference drug benznidazol (SI = 5–5.8). The sulfonamide derivatives have a great potential as anti-*T. cruzi* agents but they were slightly toxic (Table 2). The best SI was found with derivative **5C** against the epimastigote form of *T. cruzi* strain Y (SI = 5.09), which is comparable to that of the standard drug benznidazol (Table 2).

**Table 2.** IC_50_ and IC_90_ values derived from growth inhibition assays of *Trypanosoma cruzi* (DM28c, Y) and determination of cytotoxicity (CC_50_), selectivity index (SI_50_) of **3F**, **3G**, **3W**, **5B**, **5C**, **5D** NEs.

	Drug nanoemulsions						
	**3F**	**3G**	**3W**	**5B**	**5C**	**5D**	**BZN**
Tc DM28c							
IC_50_^1^ µM	3.54^a^ ± 1.53	5.66^b^ ± 1.62	7.36^b^ ± 1.54	6.24^b^ ± 0.18	3.98^a^ ± 0.24	6.69^b^ ± 1.85	20.63^c^ ± 1.831
IC_90_^2^ µM	49.56^a^ ± 9.61	84.87^b^ ± 5.16	68.64^c^ ± 23.47	84.46^b^ ± 6.80	64.34^c^ ± 6.47	120.54^d^ ± 7.89	>128
CC_50_^3^ µM	8.13^a^ ± 1.19	6.77^b^ ± 1.07	3.21^c^ ± 0.55	6.51^b^ ± 1.11	8.04^a^ ± 1.33	6.75^b^ ± 0.98	127.54^d^ ± 12.04
SI_50_^4^	2.25^a^ ± 0.17	1.20^b^ ± 0.21	0.44^c^ ± 0.12	1.08^b^ ± 0.11	2.02^a^ ± 0.22	1.05^b^ ± 0.09	5.54^d^ ± 1.82
TcY							
IC_50_^1^ µM	2.83^a^ ± 0.71	2.27^a^ ± 0.56	3.51^b^ ± 0.12	3.47^b^ ± 0.35	2.15^a^ ± 0.29	3.27^b^ ± 0.53	21.92^c^ ± 1.67
IC_90_^2^ µM	>128	83.61^a^ ± 38.25	114.97^b^ ± 8.16	82.03^a^ ± 8.01	78.04^a^ ± 32.46	52.67^c^ ± 7.70	>128
CC_50_^3^ µM	8.13^a^ ± 1.19	6.77^b^ ± 1.07	3.21^c^ ± 0.55	6.51^b^ ± 1.11	8.04^a^ ± 1.33	6.75^b^ ± 0.98	127.54^d^ ± 12.04
SI_50_^4^	2.89^a^ ± 1.02	3.09^a^ ± 1.33	0.44^b^ ± 0.06	1.95^c^ ± 0.97	5.09^d^ ± 1.41	1.76^c^ ± 0.30	5.89^d^ ± 0.81

1 Concentration in μM which reduced the proliferation of epimastigotes by 50%.

2 Concentration in μM which reduced the proliferation of epimastigotes by 90%.

3 Concentration in cytotoxic μg ml^−1^ to 50% of RAW 267.4 cells.

4 IS_50_ Selectivity index of 50% = CC_50_/IC_50_.

a,b,c,d in the rows, means followed by different letters differ statistically (*p* < .05).

### 3.3 Flow cytometry

**Figure 4.**
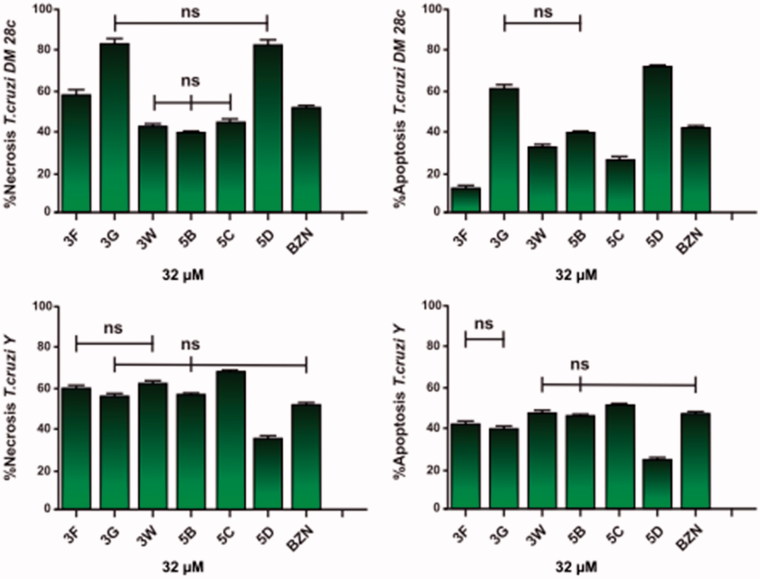
Representative graphs of flow cytometric analysis for nanoemulsions of the sulfonamides **3F, 3G, 3W, 5B, 5C**, **5D**, and benznidazole (BZN) at 32** **μΜ. (A, C) Necrosis using propidium iodide (PI) and (B, D) apoptosis using annexin V-FITC (ANV).

**Figure 5.**
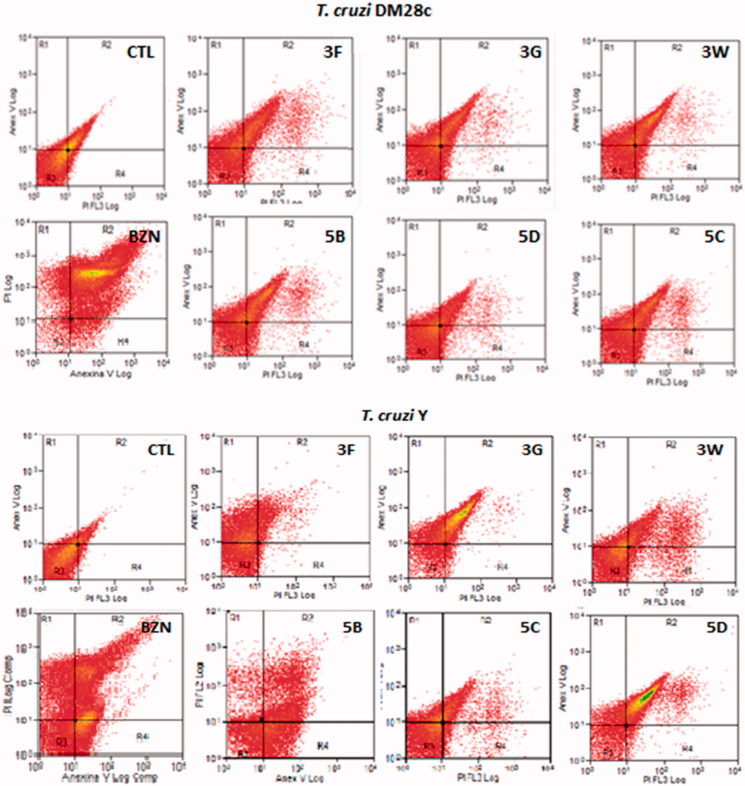
Histogram of epimastigotes representative of apoptosis analysis by flow cytometry using propidium iodide (PI) and annexin V-FITC. CTL: control without sulfonamides derivates (A) *T. cruzi* Dm28c and (B) T. cruzi Y Nanoemulsions of **3F, 3G, 3W, 5B, 5C, 5D** sulfonamides derivatives at 32 μM. R1: Cells marked with PI only (necrosis); R2: cells labelled with annexin V and PI (late apoptosis); R3: unlabelled cells (viable cells); R4: cells marked only with annexin V (early apoptosis).

Apoptosis and necrosis are different types of cell death. Apoptosis, or programed cell death, is a form of cell death that is generally triggered by normal physiological processes. On the other hand, necrosis is a premature cell death that can be caused by external factors. They can be differentiated by flow cytometry using distinct dyes. Annexin V (AV) is a marker of apoptosis, being a Ca^2+^-dependent phospholipid-binding protein with a high affinity for phosphatidylserine. Propidium iodide (PI) is a fluorescent dye necrosis indicator. It is a cell-impermeant dye that intercalates DNA and RNA of cells with damaged plasma membrane. The NEs containing the sulfonamides **3G**, **5D**, and **3F** lead the cell death by necrosis, in the following proprotions, of 82.41%, 81.26% and 57.03%, respectively for the *T. cruzi* Dm28c strain, being more effective than the reference drug benznidazole **(**effect of 51.16%). The drugs **3G**, **5B**, and **5D** induced more apoptosis than benznidazole too. Similar values were found for the *T. cruzi* Y strain. The sulfonamide NEs killed the parasites by necrosis in the proportion of 54.80% for **3G**, of 62.43% for **3W**, of 55.67% for **5B**, and of 67.01% for **5C**. These results indicate the sulfonamides in glove oil NEs were more effective in their anti *T. cruzi* effects than benznidazole. Apoptosis occurred only with **5C** in a statistically significant manner (Figure 4). In addition it was observed that most AV-positive cells were also positive for PI, suggesting that apoptotic cells evolved to secondary necrosis, with the possibility that annexin is bound to internal phosphatidylserine residues after the membrane integrity was lost (Figures 4 and 5).

## 4. Conclusions

Sulfonamide CAIs have various pharmacologic applications, as shown in the introduction of this paper. Although we have discovered low nanomolar *in vitro* TcCA inhibitors, in our first reports we could not evidence *in vivo* efficacy of such agents in interfering with the life cycle of the pathogen^20^^,^^22^. Thus, we have hypothesized that this lack of effect is due to problems of permeability of the sulfonamide through the biological membrane of the protozoan. This is the reason why we have explored the formulation of these CAIs as NEs in clove oil. The approach was in fact successful, since several sulfonamide strong TcCA inhibitors indeed showed significant anti-*T. cruzi* effects, against two different strains of the pathogen. These effects are probably due to an enhanced permeation of the enzyme inhibitor through the NE formulation, interfering in this way with the life cycle of the pathogen, either by inhibiting pH regulation or carboxylating reactions in which bicarbonate/CO_2_ are involved. This type of formulation of sulfonamides with *T. cruzi* CA inhibitory effects may lead to novel therapeutic approaches against this orphan disease.

## References

[1] Bern C. Chagas’ disease. New Engl J Med 2015;373:456–66. 26222561 10.1056/NEJMra1410150

[2] Vermelho AB, Capaci GR, Rodrigues IA, et al Carbonic anhydrases from Trypanosoma and Leishmania as anti-protozoan drug targets. Bioorg Med Chem 2017;25:1543–55. 28161253 10.1016/j.bmc.2017.01.034

[3] Correia JPR, Costa ACD, Rocha EA, et al Pharmacotherape-utic follow-up of patients with Chagas disease using benznidazole: drug-related problems and pharmaceutical interventions. Rev Soc Bras Med Trop 2017;50:334–40. 28700051 10.1590/0037-8682-0474-2016

[4] Franciso AF, Jayawardahna S, Lewis MD, et al Biological factors that impinge on Chagas disease drug development. Parasitology 2017;3:1–10. 10.1017/S0031182017001469 PMC5729846 28831944

[5] Shah P, Bhalodia D, Shelat P. Nanoemulsions: preparation, structure, functional properties and their antimicrobial effects. Sys Rev Pharm 2016;3:E378 DOI:10.4103/0975-8453.59509

[6] de Campos VE, Ricci-Júnior E, Mansur CR. Nanoemulsions as delivery systems for lipophilic drugs. J Nanosci Nanotechnol 2012;12:2881–90. 22755138 10.1166/jnn.2012.5690

[7] Teixeira HF, Bruxel F, Fraga M, et al Cationic nanoemulsions as nucleic acids delivery systems. Int J Pharm 2017;534:356–367. 29038065 10.1016/j.ijpharm.2017.10.030

[8] Araujo SC, Mattos ACA, Teixeira HF, et al Improvement of in vitro efficacy of novel schistomicidal drug by incorporation into nanoemulsions. Int J Pharm Pharmaceutical Nanotecnol 2007;337:307–315. 10.1016/j.ijpharm.2007.01.009 17292573

[9] Senna JP, Ricci-Júnior E, Mansur CRE. Development and evaluation of nanoemulsions containing phthalocyanines for use in photodynamic cancer therapy. J Nanosci Nanotechno 2015;6:4205–14. 10.1166/jnn.2015.9609 26369031

[10] Supuran CT. Carbonic anhydrases: novel therapeutic applications for inhibitors and activators. Nat Rev Drug Discov 2008;7:168–81. 18167490 10.1038/nrd2467

[11] Supuran CT. How many carbonic anhydrase inhibition mechanisms exist? J Enzyme Inhib Med Chem 2016;31:345–60. 26619898 10.3109/14756366.2015.1122001

[12] Supuran CT. Structure-based drug discovery of carbonic anhydrase inhibitors. J Enzyme Inhib Med Chem 2012;27:759–72. 22468747 10.3109/14756366.2012.672983

[13] Scozzafava A, Supuran CT, Carta F. Antiobesity carbonic anhydrase inhibitors: a literature and patent review. Expert Opin Ther Pat 2013;23:725–35. 23607332 10.1517/13543776.2013.790957

[14] (a) Nocentini A, Carta F, Ceruso M, et al Click-tailed coumarins with potent and selective inhibitory action against the tumor-associated carbonic anhydrases IX and XII Bioorg. Med Chem 2015;23:6955–66. 10.1016/j.bmc.2015.09.041 26432607

[15] Capasso C, Supuran CT. Sulfa and trimethoprim-like drugs–antimetabolites acting as carbonic anhydrase, dihydropteroate synthase and dihydrofolate reductase inhibitors. J Enzyme Inhib Med Chem 2014;29:379–87. 23627736 10.3109/14756366.2013.787422

[16] Supuran CT, Capasso C. New light on bacterial carbonic anhydrases phylogeny based on the analysis of signal peptide sequences. J Enzyme Inhib Med Chem 2016;31:1254–60. 27353388 10.1080/14756366.2016.1201479

[17] Capasso C, Supuran CT. An overview of the alpha-, beta- and gamma-carbonic anhydrases from bacteria: can bacterial carbonic anhydrases shed new light on evolution of bacteria? J Enzyme Inhib Med Chem 2015;30:325–32. 24766661 10.3109/14756366.2014.910202

[18] Capasso C, Supuran CT. Bacterial, fungal and protozoan carbonic anhydrases as drug targets. Expert Opin Ther Targets 2015;19:1689–704. 26235676 10.1517/14728222.2015.1067685

[19] (a) Diaz JR, Fernández Baldo M, Echeverría G, et al A substituted sulfonamide and its Co (II), Cu (II), and Zn (II) complexes as potential antifungal agents. J Enzyme Inhib Med Chem 2016;31: 51–62. 27232977 10.1080/14756366.2016.1187143

[20] Supuran CT. Inhibition of carbonic anhydrase from *Trypanosoma cruzi* for the management of Chagas disease: an underexplored therapeutic opportunity. Future Med Chem 2016;8:311–24. 26898220 10.4155/fmc.15.185

[21] Prete SD, Vullo D, Fisher GM, et al Discovery of a new family of carbonic anhydrases in the malaria pathogen *Plasmodium falciparum*—the η-carbonic anhydrases. Bioorg Med Chem Lett 2014;24:4389–96. 25168745 10.1016/j.bmcl.2014.08.015

[22] Supuran CT, Scozzafava A, Mastrolorenzo A. Bacterial proteases: current therapeutic use and future prospects for the development of new antibiotics. Expert Opin Ther Pat 2001;11:221–59.

[23] (a) Capasso C, Supuran CT. An overview of the selectivity and efficiency of the bacterial carbonic anhydrase inhibitors. Curr Med Chem 2015;22:2130–9. 25312213 10.2174/0929867321666141012174921

[24] Pan P, Vermelho AB, Capaci Rodrigues G, et al Cloning, characterization, and sulfonamide and thiol inhibition studies of an α-carbonic anhydrase from *Trypanosoma cruzi*, the causative agent of Chagas disease. J Med Chem 2013;56:1761–71. 23391336 10.1021/jm4000616

[25] Güzel-Akdemir Ö, Akdemir A, Pan P, et al A class of sulfonamides with strong inhibitory action against the α-carbonic anhydrase from *Trypanosoma cruzi*. J Med Chem 2013;56:5773–81. 23815159 10.1021/jm400418p

[26] de Menezes Dda R, Calvet CM, Rodrigues GC, et al Hydroxamic acid derivatives: a promising scaffold for rational compound optimization in Chagas disease. J Enzyme Inhib Med Chem 2016;31:964–73. 26327246 10.3109/14756366.2015.1077330

[27] Rodrigues GC, Feijó DF, Bozza MT, et al Design, synthesis, and evaluation of hydroxamic acid derivatives as promising agents for the management of Chagas disease. J Med Chem 2014;57:298–308. 24299463 10.1021/jm400902y

[28] Pan P, Vermelho AB, Scozzafava A, et al Anion inhibition studies of the α-carbonic anhydrase from the protozoan pathogen *Trypanosoma cruzi*, the causative agent of Chagas disease. Bioorg Med Chem 2013;21:4472–6. 23790722 10.1016/j.bmc.2013.05.058

[29] Garaj V, Puccetti L, Fasolis G, et al Carbonic anhydrase inhibitors: novel sulfonamides incorporating 1,3,5-triazine moieties as inhibitors of the cytosolic and tumour-associated carbonic anhydrase isozymes I, II and IX. Bioorg Med Chem Lett 2005;15:3102–8. 15905091 10.1016/j.bmcl.2005.04.056

[30] Zetasizer Nano User manual. Malvern: Malvern Instruments. Man0485, n. 1.1, 2004 Available from: http://www.malvern.com/en/support/resource-center/usermanuals/MAN0485EN.aspx. [last accessed 20 Nov 2014].

[31] Aymerich S, Goldenberg S. The karyotype of *Trypanosoma cruzi* Dm 28c: comparison with other *T. cruzi* strains and trypanosomatids. Experiment Parasitol 1989;69:107–15. 10.1016/0014-4894(89)90178-1 2666150

[32] Silva LHP, Nussenweig V. Sobre uma cepa de *Trypanosoma cruzi* altamente virulenta para o camundongo branco. [On a strain of *Trypanosoma cruzi* highly virulent for white mice]. V Fol Clin Biolog 1953;20:191–207.

[33] Alvarenga NJ, Bronfen E. Metaciclogênese do *Trypano-soma cruzi* como parâmetro de interação do parasita com o triatomíneo vetor. Ver Soc Bras Med Trop 1997;30:247–50. 10.1590/s0037-86821997000300012 9273572

[34] Rodrigues IA, Silva B, Santos ALS, et al A new experimental culture medium for cultivation of *Leishmania amazonensis*: its efficacy for the continuous in vitro growth and differentiation of infective promastigote forms. Parasitology Res 2010;106:1249–52. 10.1007/s00436-010-1775-4 20177905

[35] Rolon M, Vega C, Escario JA, et al Development of resazurin microtiter assay for drug sensibility testing of *Trypanosoma cruzi* epimastigotes. Parasitol Res 2006;99:103–7. 16506080 10.1007/s00436-006-0126-y

[36] Mosmann T. Rapid colorimetric assay for cellular growth and survival: application to proliferation and cytotoxicity assays. J Immunol Methods 1983;983:55–63. 10.1016/0022-1759(83)90303-4 6606682

[37] Romanha AJ, Castro SL, Soeiro MNC, et al In vitro and in vivo experimental models for drug screening and development for Chagas disease. Mem Inst Oswaldo Cruz 2010;105:233–8. 20428688 10.1590/s0074-02762010000200022

